# The 2023 Impact of Inflammatory Bowel Disease in Canada: Mental Health and Inflammatory Bowel Disease

**DOI:** 10.1093/jcag/gwad012

**Published:** 2023-09-05

**Authors:** Lesley A Graff, Rose Geist, M Ellen Kuenzig, Eric I Benchimol, Gilaad G Kaplan, Joseph W Windsor, Alain Bitton, Stephanie Coward, Jennifer L Jones, Kate Lee, Sanjay K Murthy, Juan-Nicolás Peña-Sánchez, Laura E Targownik, Nazanin Jannati, Tyrel Jones May, Tasbeen Akhtar Sheekha, Tal Davis, Jake Weinstein, Ghaida Dahlwi, James H B Im, Jessica Amankwah Osei, Noelle Rohatinsky, Sara Ghandeharian, Quinn Goddard, Julia Gorospe, Shira Gertsman, Michelle Louis, Richelle Wagner, Colten Brass, Rhonda Sanderson, Charles N Bernstein

**Affiliations:** Department of Clinical Health Psychology, Max Rady College of Medicine, Rady Faculty of Health Sciences, University of Manitoba, Winnipeg, Manitoba, Canada; University of Manitoba IBD Clinical and Research Centre, Winnipeg, Manitoba, Canada; Department of Psychiatry, The Hospital for Sick Children, University of Toronto, Toronto, Canada; SickKids Inflammatory Bowel Disease Centre, Division of Gastroenterology, Hepatology, and Nutrition, The Hospital for Sick Children, Toronto, Ontario, Canada; Child Health Evaluative Sciences, SickKids Research Institute, The Hospital for Sick Children, Toronto, Ontario, Canada; SickKids Inflammatory Bowel Disease Centre, Division of Gastroenterology, Hepatology, and Nutrition, The Hospital for Sick Children, Toronto, Ontario, Canada; Child Health Evaluative Sciences, SickKids Research Institute, The Hospital for Sick Children, Toronto, Ontario, Canada; ICES, Toronto, Ontario, Canada; Department of Paediatrics, Temerty Faculty of Medicine, University of Toronto, Toronto, Ontario, Canada; Institute of Health Policy, Management, and Evaluation, Dalla Lana School of Public Health, University of Toronto, Toronto, Ontario, Canada; Departments of Medicine and Community Health Sciences, University of Calgary, Calgary, Alberta, Canada; Departments of Medicine and Community Health Sciences, University of Calgary, Calgary, Alberta, Canada; Division of Gastroenterology and Hepatology, McGill University Health Centre IBD Centre, McGill University, Montréal, Quebec, Canada; Departments of Medicine and Community Health Sciences, University of Calgary, Calgary, Alberta, Canada; Departments of Medicine, Clinical Health, and Epidemiology, Dalhousie University, Halifax, Nova Scotia, Canada; Crohn’s and Colitis Canada, Toronto, Ontario, Canada; Department of Medicine, University of Ottawa, Ottawa, Ontario, Canada; The Ottawa Hospital IBD Centre, Ottawa, Ontario, Canada; Department of Community Health and Epidemiology, University of Saskatchewan, Saskatoon, Saskatchewan, Canada; Division of Gastroenterology and Hepatology, Mount Sinai Hospital, University of Toronto, Toronto, Ontario, Canada; Department of Community Health and Epidemiology, University of Saskatchewan, Saskatoon, Saskatchewan, Canada; Division of Gastroenterology and Hepatology, University Health Network, University of Toronto, Toronto, Ontario, Canada; Department of Community Health and Epidemiology, University of Saskatchewan, Saskatoon, Saskatchewan, Canada; SickKids Inflammatory Bowel Disease Centre, Division of Gastroenterology, Hepatology, and Nutrition, The Hospital for Sick Children, Toronto, Ontario, Canada; Child Health Evaluative Sciences, SickKids Research Institute, The Hospital for Sick Children, Toronto, Ontario, Canada; SickKids Inflammatory Bowel Disease Centre, Division of Gastroenterology, Hepatology, and Nutrition, The Hospital for Sick Children, Toronto, Ontario, Canada; Child Health Evaluative Sciences, SickKids Research Institute, The Hospital for Sick Children, Toronto, Ontario, Canada; SickKids Inflammatory Bowel Disease Centre, Division of Gastroenterology, Hepatology, and Nutrition, The Hospital for Sick Children, Toronto, Ontario, Canada; Department of Pediatrics, Faculty of Medicine, King Abdulaziz University, Jeddah, Saudi Arabia; SickKids Inflammatory Bowel Disease Centre, Division of Gastroenterology, Hepatology, and Nutrition, The Hospital for Sick Children, Toronto, Ontario, Canada; Child Health Evaluative Sciences, SickKids Research Institute, The Hospital for Sick Children, Toronto, Ontario, Canada; Department of Community Health and Epidemiology, University of Saskatchewan, Saskatoon, Saskatchewan, Canada; College of Nursing, University of Saskatchewan, Saskatoon, Saskatchewan, Canada; Crohn’s and Colitis Canada, Toronto, Ontario, Canada; Departments of Medicine and Community Health Sciences, University of Calgary, Calgary, Alberta, Canada; Departments of Medicine and Community Health Sciences, University of Calgary, Calgary, Alberta, Canada; Michael G. DeGroote School of Medicine, McMaster University, Hamilton, Ontario, Canada; Crohn’s and Colitis Canada, Toronto, Ontario, Canada; Department of Educational Psychology, University of Alberta, Edmonton, Alberta, Canada; Muskoday First Nation, Saskatchewan, Canada; James Smith Cree Nation, Saskatchewan, Canada; University of Manitoba IBD Clinical and Research Centre, Winnipeg, Manitoba, Canada; Department of Internal Medicine, Max Rady College of Medicine, Rady Faculty of Health Sciences, University of Manitoba, Winnipeg, Manitoba, Canada

**Keywords:** Anxiety, Crohn’s disease, Depression, Mental health, Ulcerative colitis

## Abstract

Psychiatric disorders are 1.5 to 2 times more prevalent in persons with inflammatory bowel disease (IBD) than in the general population, with pooled prevalence estimates of 21% for clinical anxiety and 15% for depression. Rates are even higher when considering mental health symptoms, as nearly one-third of persons with IBD experience elevated anxiety symptoms and one-quarter experience depression symptoms. Rates of these symptoms were much higher during periods of disease activity, more common in women than men, and more common in Crohn’s disease than ulcerative colitis. There is robust evidence of the detrimental effects of comorbid depression and anxiety on the subsequent course of IBD based on longitudinal studies tracking outcomes over time. However, psychiatric disorders and IBD have bidirectional effects, with each affecting risk of the other. Elevated mental health concerns have been consistently associated with greater healthcare utilization and costs related to IBD. There is some signal that low resilience in adolescence could be a risk factor for developing IBD and that enhancing resilience may improve mental health and intestinal disease outcomes in IBD. Psychological therapies used to treat anxiety and depression occurring in the context of IBD have been shown to significantly improve the quality of life for persons with IBD and reduce anxiety and depression. There is less evidence in regard to the impact of psychotropic medications on mental health or disease outcomes in persons with IBD. There is consensus, however, that mental health must be addressed as part of comprehensive IBD care for children and adults.

**Key Points**

1. Psychiatric disorders are 1.5–2 times more prevalent in persons with IBD than the general population, with prevalence rates of nearly 21% for diagnosed anxiety and 15% for depression, and yet they can go undetected. Youth with IBD have nearly double the risk of a psychiatric diagnosis, and sixfold the risk of depression.
2. Nearly one-third of persons with IBD experience ­elevated anxiety symptoms and one-quarter experience depression symptoms, with higher rates in women and persons with Crohn’s disease.
3. Depression and anxiety adversely affect the disease course of IBD and increase associated healthcare utilization; active IBD can adversely affect mental health.
4. Psychological therapies used to treat anxiety and depression occurring in the context of IBD have been shown to significantly improve quality of life and reduce anxiety and depression for children and adults with IBD; there is less direct evidence in regard to the impact of antidepressant medication on mental health or disease outcomes in IBD.
5. High resilience is associated with improved mental health and IBD outcomes and is a promising target for intervention.
6. Strengthening autonomy in disease self-management may be especially important for adolescents with IBD to facilitate transition from pediatric to adult care.
7. Clinical guidelines recommend screening people with IBD for mental health concerns as part of standard practice in IBD care, recognizing the prominent comorbidity and disease influence, but also noting the importance of having clinical pathways for care if positive.
8. Multidisciplinary IBD clinics, which include mental health specialists, facilitate integrated medical and psychological care and are the recommended model for children and adults with IBD.

## Summary of Crohn’s and Colitis Canada’s 2018 Impact of IBD Concerning Mental Health

In the 2018 report, mental health in relation to inflammatory bowel disease (IBD) was interwoven throughout several chapters. The 2018 report identified the broad reach of mental health on multiple aspects of IBD including symptom expression, disease course, response to advanced therapies, and severe outcomes such as hospitalization and death. Psychological distress was reported to be elevated not just during periods of active disease but also during remission. Other psychosocial factors that were relevant for individuals with IBD included body image concerns, fear of sexual inadequacy, worry about dependency and stigmatization, social isolation, and concern about not reaching one’s full potential. Mental illnesses were reported to be twice as common for those with IBD compared to the general community and thought to be more common than any other extraintestinal conditions associated with IBD. It was notable that not that many years ago, discussion of extraintestinal diseases of IBD would have only examined the musculoskeletal system, skin, eyes, and biliary tract. An important theme was the need for multidisciplinary care for those with IBD, integrating care from mental health specialists to address mental health concerns and overall disease management.

## Risk of Mental Health Disorders and Symptoms in IBD

IBD is a complex, chronic condition with fluctuating and often unpredictable disease course. There are challenges not only in treating gastrointestinal and extraintestinal manifestations of disease but also in addressing psychological distress and mental health concerns. This review focuses predominantly on comorbid anxiety and depression in IBD, as these are the most common mental health presentations. There are other mental illnesses emerging as potentially relevant for IBD, such as disease-related post-traumatic stress, substance misuse, and eating disorders, which are beyond the scope of this review, but may be addressed in a future impact report as further research becomes available.

One in five Canadians experience a mental illness in any given year (1); this rate is even higher for adults with IBD. Epidemiologic studies have found that individuals with IBD have a higher incidence of diagnosed mental illnesses compared to matched controls, inclusive of depression (18.6 per 1,000 people; 95% CI: 14.2, 24.3 vs. 10.8 per 1,000; 95% CI: 9.26, 12.5), anxiety (25.0 per 1,000; 95% CI: 19.6, 32.0 vs. 16.3 per 1,000; 95% CI: 14.3, 18.6), bipolar illness (3.8 per 1,000; 95% CI: 2.29, 6.30 vs. 1.56 per 1,000; 95% CI: 1.09, 2.23), and schizophrenia (4.62 per 1,000; 95% CI: 3.12, 6.84 vs. 3.15 per 1,000; 95% CI: 2.53, 3.92), as well as higher prevalence of these mental health conditions overall (2,3). The pooled prevalence of clinical anxiety was 20.5% (4.9%, 36.5%) and depression was 15% (9.9%, 20.5%) in a systematic review of 171 studies including over 158,000 participants with IBD (4). The highest risk for a mental health concern has been seen in the first year after IBD diagnosis (hazard ratio [HR]: 1.4; 95% CI: 1.2, 1.6) (5), however, depression or anxiety can present at any time in the course of the illness. In addition, while still very uncommon, adults with IBD have a higher risk of suicidality compared to the general community (HR: 1.2, 1.4) (5). Even with this higher risk for these mental health concerns, depression may be undetected in one-third, and anxiety undetected in two-thirds of people with IBD (6).

The pattern is similar when considering symptom rates of comorbid anxiety or depression in IBD. A recent systematic review and meta-analysis of 77 studies including over 30,000 individuals with IBD indicated that the pooled prevalence was almost twice as high as the general community, with close to one in three with IBD experiencing elevated anxiety symptoms (32.1%; 95% CI: 28.3%, 36.0%), and one in four with IBD experiencing depression (25.2%; 95% CI: 22.0%, 28.5%) (7). Further, rates were as high as 58% and 39%, respectively, during periods of disease activity, and symptoms of anxiety and depression were more common for women than men, and for those with Crohn’s disease compared with ulcerative colitis (7).

Childhood and adolescence generally are periods of risk for the development of psychiatric disorders, with prevalence estimates as high as 20% (8). Children with chronic diseases have an increased prevalence and impact of mental health conditions (9), and young people with psychiatric disorders, especially depression, can have impairments in social and educational functioning (10). A systematic review of psychiatric disorders in IBD, which included pediatric (nine studies), adult (36 studies) and both adult and pediatric participants (24 studies), found higher rates of mental disorders for children with IBD as well as for adults—most commonly anxiety and depression (11). A meta-analytic review of studies comparing youth with IBD to those with other chronic illnesses reported similar rates of anxiety disorders (OR: 1.90; 95% CI: 0.47, 7.69; *P* = 0.37), and higher risk of depressive disorders for youth with IBD (OR: 5.80; 95% CI: 1.60, 21.03; *P* = 0.007) (12).

## Mechanisms of Comorbid Mental Health Disorders and IBD

Reviews of depression and anxiety suggest inflammation and microbiota play a role in the development of these disorders, which may provide explanatory pathways relevant to IBD (13,14). More specifically, a study of mice with induced colitis found that chronic stress was associated with a significant increase in inflammation-enhancing bacteria (15). Interestingly, higher rates of mental disorders relative to matched controls have been found to pre-date an IBD diagnosis by up to five years, suggesting bidirectional vulnerability (16). Corticosteroid medications used in the care of IBD, such as prednisone, have well-known mood disturbance effects (17). However, there has been surprisingly little examination of adverse psychiatric events with biologics, despite their increasingly widespread use for IBD over the past 20 years. A recent systematic review and meta-analysis found only 15 reported incidences of an adverse psychiatric event for over 2,600 individuals with IBD receiving any of the currently available biologics, based on eligible randomized controlled trials (18). The study concluded that there was not sufficient evidence of any greater risk of depression, anxiety, psychosis, or suicide associated with the use of biologics, reporting a pooled risk difference of 0.01 (95% CI: 0.00, 0.02) comparing those with IBD on biologics to those not taking biologics.

## The Bidirectional Influence of Mental Health and Disease Course in IBD

There is robust support for the detrimental effects of depression and anxiety on the subsequent course of IBD, based on longitudinal studies tracking outcomes over time (19). The Swiss IBD Cohort Study (*n* = 1,973), which evaluated depression annually, found that elevated depressive symptoms were strong risk factors for multiple adverse disease outcomes, including recurrence of active disease (adjusted hazard ratio [aHR]: 3.55; 95% CI: 2.34, 5.39), a new fistula (aHR: 1.81; 95% CI: 1.03, 3.17), having surgery (aHR: 2.16; 95% CI: 1.48, 3.17), experiencing primary nonresponse to therapy (aHR: 1.91; 95% CI: 1.10, 3.31), or having systemic corticosteroid therapy (aHR: 1.58; 95% CI: 1.06, 2.37), with a higher risk overall for clinical deterioration (20). In addition, elevated depressive symptoms were associated with the onset of new extraintestinal manifestations, including peripheral arthritis/arthralgia (aHR: 1.42; 95% CI: 1.02, 1.98), primary sclerosing cholangitis (aHR: 3.97; 95% CI: 1.35, 11.68), and uveitis/iritis (aHR: 2.24; 95% CI: 1.17, 4.29) (20).

In a study from Manitoba, Canada, which followed an IBD cohort of 247 individuals over three years, the risk of subsequent active IBD was increased sixfold with elevated depressive symptoms (OR: 6.27; 95% CI: 1.39, 28.2), and elevated symptoms of anxiety doubled the risk for active IBD (OR: 2.17; 95% CI: 1.01, 4.66) (21). Further, a two-year follow-up study of 228 individuals in the United Kingdom with established IBD remission at baseline had a significantly higher risk of disease flare and escalation of medication therapy if they had elevated psychological comorbidities at baseline (HR: 3.18; 95% CI: 1.44, 7.02; HR: 2.48; 95% CI: 1.03, 5.93, respectively) (22).

While many of the disease outcomes reviewed in conjunction with mental health comorbidity are objective, such as medication use and fistula development, few studies have explored the relationship between mental health symptoms and specific markers of inflammation. In a Canadian cross-sectional study, individual-reported high stress was associated with increased disease symptoms but not with concurrent fecal calprotectin (23). The association between gastrointestinal symptoms and fecal calprotectin is modest at best (24,25), and may require closer longitudinal tracking.

Depression and anxiety have also been implicated in lower cognitive functioning for individuals with IBD. Higher rates of impairment in processing speed, verbal learning, and working memory, using validated cognitive tests, were found for those with IBD compared to population norms (25). Higher levels of anxiety were significantly associated with reduced processing speed and verbal learning (*P* < 0.001), and higher levels of depression symptoms were associated with slower processing speed (*P* < 0.01) in the IBD sample of 247 participants (25), suggesting that managing symptoms of anxiety and depression in IBD may also be important to mitigate their effect on cognitive functioning.

Overall, the presence of these mental health symptoms appears to have a role in a poorer disease course for IBD, and having IBD may in turn impact the risk of developing depression or anxiety. Individuals with pediatric-onset IBD (mean age 14 years, *n* = 6,464) had a twofold increased risk for any psychiatric disorder, inclusive of mood, anxiety, and eating disorders, over a nine-year period when compared to matched controls (*n* = 323,200); further, mental health disorders were more common for those with very early onset of IBD (onset younger than age six) (26). These findings were very similar to those observed in a study using the Taiwanese National Health Insurance Research Database, where 18.5% of people with IBD developed depression during the 11-year study compared to only 4.8% in non-IBD siblings (adjusted odds ratio [aOR]: 9.43; 95% CI: 6.43, 13.81) and 2.5% for matched controls (aOR: 1.82; 95% CI: 1.14, 2.91) (27). Another study of 405 persons with IBD who were followed for over two years found an almost sixfold increase in risk for subsequent significant anxiety for those who had active Crohn’s disease or ulcerative colitis at baseline (HR: 5.77; 95% CI: 1.89, 17.7) (28).

## Mental Health Disorders and Healthcare Utilization in IBD

The high cost of mental health challenges in the context of IBD is not only reflected in the psychological distress experienced by the individual, and the impact on the course of the IBD, but also on health system utilization. That is, with the adverse impact of these mental health presentations on IBD, it is not surprising that there is also evidence of greater IBD-related healthcare needs for people with IBD who have mental health comorbidities.

Both retrospective and prospective cohort studies, ranging in sample size from 400 to over 300,000, and following individuals with IBD who had elevated depression and anxiety found a significantly greater likelihood of disease relapse, hospitalization, emergency department (ED) visits, and higher healthcare costs (Table 1) (29–34). A study utilizing provincial administrative health databases also reported higher levels of physician visits and hospital length of stay for those with IBD and comorbid mental health disorders (anxiety, depression, bipolar disorder) relative to matched controls, even after accounting for those who were directly mental health related (32). Similarly, a study of Canadian children with IBD found that mental health diagnosis was one of the strongest predictors of having high direct healthcare costs in the first year after IBD diagnosis (35).

**Table 1.** Impact of mental health concerns on healthcare utilization in IBD

Study characteristics	Region	Type of study	Sample size	outcomes
Anxiety and depression at baseline, adjusted for multiple variables including severity of disease at baseline and prior IBD-related surgeries; followed four years (29).	Canada	Prospective cohort two tertiary GI referral centres	414 (IBD)	Anxiety: risk factor for poorer IBD outcomes defined as IBD-related ED visits, IBD-related hospitalization, or two or more courses of systemic steroids within one year (OR: 3.36; 95% CI: 1.51, 7.48) Depression: aOR not significant; however only 4% of the sample had elevated depressive symptoms at baseline, suggesting potential floor effects.
Depression at baseline adjusted for sex, disease status; followed two years (30).	United States	Prospective cohort seven tertiary IBD referral centres	4,314 (IBD)	CD: increased risk for disease relapse (RR: 2.3; 95% CI: 1.9, 2.8), surgery or hospitalization (RR: 1.3; 95% CI: 1.1, 1.6); UC: increased risk for surgery or hospitalization (RR: 1.3; 95% CI: 1.1, 1.5).
Anxiety and depression at initial encounter; study period 20 months (31).	United States	Retrospective cohort Tertiary IBD referral centre	432 (IBD)	Higher rates of utilization for comorbid anxiety, depression compared to IBD only: Imaging studies (53.6% vs. 36.7%, *P* < 0.05), ED visits (30.7% vs. 20.8%, *P* < 0.05) Hospitalized (31.7% vs. 21.7%, *P* < 0.05), Prescribed corticosteroids (50.5% vs. 36.7%, *P* < 0.01) Prescribed biologic medications (62.5% vs. 51.3%, *P* < 0.05).
Comorbid anxiety, depression, bipolar disorder using validated case definitions (32).	Canada	Retrospective cohort Provincial health administrative database	8,459 (IBD), 40,375 (matched controls)	Higher rates of utilization for those with IBD comorbid psychiatric disorders: Active psychiatric comorbidity was associated with >10 more physician visits, 3.1 more hospital days, used >6.3 more drugs. There was a synergistic effect of IBD (vs. no IBD) and psychiatric comorbidity (vs. no psychiatric comorbidity). Higher rates remained, after accounting for mental health-related healthcare utilization
IBD hospitalization during six-month period; evaluated for comorbid anxiety, depression, bipolar disorder; followed for up to 10 months (33).	United States	Retrospective cohort: Nationwide Readmissions Database	40,177 (IBD)	Higher utilization and costs for comorbid psychiatric disorders compared to IBD only: Hospital days (median 7 days vs. 5 days, *P* < 0.01), Readmission rates—30-day (31.3 vs. 25.4%; *P* < 0.01); 90-day (42.6 vs. 35.3%; *P* < 0.01) Hospitalization-related costs (median $41,418 vs. $39,242, *P* < 0.01). Risk of readmission (HR: 1.16; 95% CI: 1.13, 1.20) Risk of severe IBD-related hospitalization (HR: 1.13; 95% CI: 1.08, 1.16).
Comorbid depression (34).	United States	Retrospective cohort, National health administrative claims database	331,772 (IBD)	Higher utilization and costs for comorbid depression compared to IBD only: IBD-related healthcare costs mean annual $17,706 (95% CI: $16,892, 18,521) ED visits (aIRR: 1.5; 95% CI: 1.5, 1.6) In the subset of IBD patients with ED visits or hospitalized, higher likelihood of: Repeated CT scans [1–4 scans] (aOR: 1.6; 95% CI: 1.5, 1.7) IBD-related surgery (aOR: 1.2; 95% CI: 1.1, 1.2).LATED

Abbreviations: aIRR, Adjusted incidence rate ratio; aOR, Adjusted odds ratio; CD, Crohns disease; CI, Confidence interval; ED, Emergency department; IBD, Inflammatory bowel disease; OR, Odds ratio; RR, Relative risk; UC, Ulcerative colitis.

## Resilience and Coping in IBD

Much of the work on mental health in IBD has focused on the illness end of the mental health continuum, with the examination of elevated distress, depression, and anxiety. An emerging area of investigation in chronic disease more generally, and specifically in IBD, shifts the emphasis to wellness, with exploration of disease adaptation aspects such as resilience and self-efficacy for disease management. Resilience is described variously as an ability or process to maintain mental health despite experience with physical or psychological adversity (36). Resilience research often considers protective mechanisms against stress-related disorders and chronic disease impact, with resiliency-based interventions aiming to prevent or mitigate mental disorders through enhancing resilience capacity.

Low resilience in adolescence could be a risk factor for developing IBD as an adult. A study of close to 240,000 young men evaluated for resiliency through mandatory military conscription in Sweden and followed for an average of 25 years found that those with low resilience in late adolescence were more likely to develop IBD (Crohn’s disease, HR: 1.39; 95% CI: 1.13, 1.71; ulcerative colitis, HR: 1.19; 95% CI: 1.03, 1.37), adjusting for any early indicators of disease (37). With the magnitude being relatively small, it is speculated that this factor may not be directly responsible for the pathogenesis of the disease, but may influence a shift from subclinical potential to clinical disease. The applicability of these findings to women is unknown.

Resilience has also been identified as a potential mediating variable in IBD. In a cross-sectional study, experiences of childhood trauma were associated with lower resilience, which in turn was related to higher depression and higher suicide risk in a sample of 172 adults with IBD (38). Given the high prevalence of adverse childhood experiences overall (39), and in persons with IBD (40), resilience, in addition to depression, may be an important target for intervention in those with IBD.

High resilience, as measured by the Connor Davidson Resilience Scale, in a sample of 288 adults with IBD, was associated with significantly lower levels of anxiety (*r*: −0.47; 95% CI: −0.58, −0.34) and depression (*r*: −0.53; 95% CI: −0.62, −0.42) (41). Anxiety remained independently associated with resilience after controlling for depression (*P* = 0.009), although the reverse was not supported for depression. Individual differences in related aspects of psychological adjustment, examining self-efficacy (confidence in ability to manage) and sense of coherence (sense of personal resources to manage), were evaluated in a sample of 299 adults with IBD (42). Lower self-efficacy and sense of coherence were significantly related to higher anxiety (*P* = 0.001) and depression (*P* = 0.02). In addition, higher resilience was found to be independently associated with lower disease activity and better quality of life for Crohn’s disease (*P* < 0.001; *P* = 0.016) and ulcerative colitis (*P* = 0.035; *P* = 0.016), respectively (43). Higher resilience was also independently associated with fewer surgeries for people with Crohn’s disease (OR: 0.127; 95% CI: 0.03, 0.45).

Very few studies to date have evaluated resilience and self-efficacy over time in IBD, despite the value of this approach to establish directionality. Findings support a potential protective mechanism of psychological adaptation, with those with higher resilience experiencing fewer depressive symptoms (44). Impact on IBD outcomes has also been promising. The Manitoba Living with IBD study monitored 154 persons with IBD for a year through biweekly validated measures completed online. After adjusting for demographic variables, higher self-efficacy was associated with lower likelihood of flare, both via self-report (OR: 0.80; 95% CI: 0.71, 0.91) and based on a validated clinical index IBDSI (OR: 0.89; 95% CI: 0.80, 0.99) (45).

## Pediatric Considerations and Mental Health

Adolescence is a period of active growth and development. Onset of IBD in childhood or adolescence can impact physical and mental health as well as family relationships. Personality development in this period includes the evolution of a sense of self, personal identity, social comfort and relationships, and the further progression of independence and autonomy (46). For the young individual with IBD, challenges in pain management and disease control can interfere with school attendance and participation in social activities, both of which are major environments for growth of academic and social skills (47). While it was found in a population-based Manitoba study that children with IBD had similar Grade 12 education outcomes as matched controls comparing aspects such as standardized test scores for English and mathematics, poorer educational outcomes were independently predicted by lower socioeconomic status and diagnosis of mental health concerns six months prior to and six months post-IBD diagnosis (48).

Natural efforts toward autonomy at this developmental stage can be undermined by the unpredictable nature of the IBD and significant medical care needs such as surgery. Parents are faced with the challenge of helping their children develop autonomy and independence while at the same time supporting or leading difficult decisions associated with their child’s medical care.

This issue is especially relevant during the period of transition around age 17 from pediatric to adult IBD care. Healthcare teams have become increasingly aware that this transition phase can result in increased anxiety, depression, and clinical symptoms and they have identified program initiatives that may facilitate this care transition period (49). For example, transition to adult care may be smoother if autonomy was actively encouraged early in the course of illness through aspects such as directly facilitating the adolescent’s knowledge and responsibility for self-management. From an early point of care, this might involve the healthcare team adopting a strategy at each visit of speaking first to the individual (age 12 or older), and then to the parents and individual together. Similarly, more directly involving the individual in care decisions and disease management can engage them as an active member of the care team. Further, it may be helpful for the team to address with parents, as the primary caregivers, the challenges of autonomy faced by their child in the context of this chronic illness. Parents of a child with a complex chronic disease have a central role in shared disease management, gradually fostering more responsibility for knowledge and decision-making by their adolescent, which can enhance that individual’s disease self-efficacy, confidence, coping, and autonomy (50). The period of transition from pediatric to adult care is reviewed in more detail in El-Matary et al. (this volume).

## Mental Health Interventions in IBD

There are well-established treatments for primary depression and anxiety in the general population, which include psychological therapies, and, in particular, targeted cognitive behavioral therapy (CBT) (51–53), as well as antidepressant medication (52), the latter with some recent controversy regarding strength of efficacy (54–56). However, empirical evaluation is important to confirm applicability and effectiveness of these therapies for mental health concerns co-occurring in the context of IBD. This is necessary because, for example, depression phenotypes with differing symptom expression have been implicated through work with adolescents with IBD (57), and gut inflammation may impact mental health and therapy response given the role of the brain-gut axis in pathobiology of IBD (58).

Clinical guidelines recommend screening individuals with IBD for mental health concerns, recognizing the prominent comorbidity and disease influence, but also noting the importance of having clinical pathways for care if positive (59,60). Psychological therapies used to treat anxiety and depression occurring in the context of IBD (predominantly CBT, medical hypnotherapy and more recently, mindfulness therapy) have been shown to significantly improve quality of life for people with IBD and reduce anxiety and depression (61–63). CBT has demonstrated efficacy for improved mental health outcomes in IBD in both pre–post (significantly reduced anxiety, depression symptoms *P* < 0.001) (64), and RCT studies, with the latter utilizing an IBD-tailored CBT protocol and finding significant benefit for moderate-to-severe mental health presentations (depression, *d* = 0.48; anxiety, *d* = 0.58) (65). A controlled mindfulness trial for people with IBD, using a group-based treatment protocol over eight sessions compared to treatment as usual, also found significant improvement in measures of anxiety, depression, and quality of life for those engaged in the mindfulness program. Immediate post-treatment effect sizes ranging from medium to large (*d* = 0.56, 1.27), and durable effects at six months post-treatment (*d* = 0.45 to 1.38) were found (66). While these therapies are usually delivered over multiple weekly sessions, results of a pilot study delivering a one-day behavioural intervention workshop, which included elements of CBT and mindfulness, showed signal for improved anxiety and depression symptoms at three months post-treatment (*P* < 0.01, *P* = 0.06, respectively); although this study was hampered by a small sample size (67).

Emerging data suggest that psychological therapies may have positive effects on IBD outcomes, prolonging remission for adults with ulcerative colitis (68,69) and potentially through reducing inflammatory responses (70), although other studies have not found improved disease outcomes after an extended time (71). Virtual health technologies, including web-based delivery of psychological therapies, most commonly CBT, have also shown promise in IBD to improve disease outcomes (72) and address mental health concerns (73). Importantly, they have the potential to broaden access to these behavioural interventions to facilitate disease self-management (72), and participants have indicated receptivity to this approach (74).

Up to 30% of adults with IBD have taken antidepressant medications, most commonly for pain, comorbid depression, and/or anxiety (75). Antidepressants were more likely to be initiated in the first year following IBD diagnosis (76), which is not surprising in light of the higher risk of mental health concerns in that time period. However, up to two-thirds of those on antidepressants discontinued the medication much earlier than the standard treatment duration, with many taking the medications less than a month; young adults were most prone to discontinue early (76). In one of the only prospective studies of antidepressant use in IBD, individuals with Crohn’s disease and depression who were using a selective serotonin receptor inhibitor or selective noradrenaline receptor inhibitor experienced improvement in anxiety and depression symptoms at six months (*p* < 0.001) as well as improved disease clinical index scores (*P* = 0.01) (77). While antidepressant medications may have beneficial effects for depression and anxiety occurring in the context of IBD, there is a need for further evaluation as higher inflammation has been found to hamper response to antidepressant medication (78), and there remains little data on efficacy directly in IBD (56,79).

Strengthening resilience may be another potential target of intervention to improve coping and IBD disease outcomes. This type of intervention is often utilized for pre-clinical or early clinical presentations such as mild depression or elevated stress, with encouragement to direct the individual with IBD to specific depression or anxiety treatment if there is a more significant clinical presentation (80). A meta-analysis, based on 11 RCTs of resilience training approaches utilized more generally in community and IBD samples, reported moderate positive effects favouring resilience training (standard mean difference [SMD]: 0.44; 95% CI: 0.23, 0.64) to improve psychological resilience (SMD: 0.58; 95% CI: 0.27, 0.89), with durable effects at 6-month follow up (SMD: 0.76; 95% CI: −0.04, 1.55) (81). Most of the studies used elements from CBT and mindfulness.

More specifically for IBD, Keefer et al. have developed a resilience analytic tool evaluating five areas (medical, nutritional, psychological, disease self-management skills, and health system access), and a resilience-based IBD care program, collectively referred to as the Gaining Resilience Through Transition (GRITT) method (82). Individuals with IBD scoring low on the GRITT resilience measure (*n* = 184) were offered a program tailored to the elements that needed strengthening, provided through an integrated care team, with outcomes compared pre/post intervention, and to a group of people with IBD who were nonparticipants (*n* = 210). Participants had eight sessions on average, most commonly behavioral health (CBT, hypnotherapy) and nutrition. Attrition rates were very modest (13%). There was significant improvement in resilience scores for GRITT participants (pre mean 46.3, post 73.6; *P* < 0.001), with a large effect size (*d* = 2.4, *P* < 0.001), a significant decrease in ED visits compared to the year before (pre = 138 visits, post = 40 visits), and a significant decrease in hospitalizations (pre = 72, post = 4). Nonparticipants had no change in ED visits compared to the prior year and had an increase in hospitalizations, noting the limitation that the study used an uncontrolled comparator sample.

Acceptance and commitment therapy (ACT) is a third-wave psychological therapy grounded in cognitive, behavioural, and mindfulness strategies, which aims to decrease stress, improve psychological flexibility, and strengthen values-based action in day-to-day life (83). While ACT has shown some promise of improved stress and quality of life in people with chronic diseases and similar efficacy as standard CBT (84,85), there has been little application with IBD to date. An RCT in adults who had mild or quiescent IBD demonstrated significant stress reduction compared to a treatment as usual control group (86), and further feasibility studies are underway (87,88). However, efficacy for anxiety, depression, resilience, or IBD clinical outcomes has yet to be examined.

## Mental Health Interventions in Pediatric IBD

Addressing mental health care in children and adolescents with IBD has been consistently identified as an integral part of IBD care (89). Collaborative, integrated models of care for mental health in pediatrics may improve access to mental health support (90). For the IBD team, where each provider is recognized as an expert who contributes to the overall treatment, mental health management may, to a large extent, be provided by members of the inter-professional, multidisciplinary care team. A first step is to screen for mental health and psychosocial concerns prior to or during the clinic visit. Issues for the individual may include family-related stress, emotional concerns, and stressors at school, in addition to the potential presence of a psychiatric disorder.

IBD care team meetings, which may include nurses, specialist physicians, nutrition consultants, and mental health professionals, can be utilized to identify and initiate a management plan that addresses co-occurring mental health needs of the individual with IBD and their family. This collaborative form of team management for mental health is an area of increasing clinical and academic interest and may help alleviate some of the resource allocation concerns inherent in the underfunding of the mental health care system (91,92). However, these care models are often lacking in pediatric centres and private practice clinics (93).

If suspected, major psychiatric issues can be assessed in consultation with a psychologist or psychiatrist, and, as noted previously, psychosocial management is often supported by several members of the team. This team may also include transition navigators with training in psychosocial support for individuals with chronic disease. However, even these supports can be unavailable to the healthcare team due to inadequate resource allocation, and mental health aspects may need to be addressed directly by the clinic physicians and nurses with consultative support.

Treatment options for a child or adolescent with IBD who is diagnosed with anxiety or depression can include psychotherapy such as CBT and medications like selective serotonin reuptake inhibitors (SSRIs) (94). Psychological therapies for comorbid mental health concerns in IBD have been effective for adolescents in particular, demonstrating improved mental health outcomes. Szigethy et al. found that depression was significantly decreased following a course of CBT (95), with treatment gains maintained at 6 months and 1 year (96). A small pilot study evaluating CBT and clinical anxiety for adolescents with IBD reported that 50% of the participants no longer met criteria for the anxiety disorder following treatment (97). CBT has also been a useful intervention more generally for children with IBD; this was shown in a large RCT that involved children and their parents in a CBT intervention demonstrating improved quality of life, decreased school absences, and more adaptive coping, as well as preliminary support for decreased flares (98).

With regard to medication approaches, although approved for the treatment of mental health disorders in youth, the optimal dose and duration of SSRIs for depression and anxiety in IBD remain uncertain (79), adherence to medication can be challenging (76), and outcomes for mental health symptoms or IBD disease course have not yet been well-established (56). The specific SSRI choice is based on the FDA recommendations for treatment of mental health disorders in youth and depends on the mental health condition being treated. Common therapeutic options include fluoxetine, sertraline, citalopram, and fluvoxamine (99–102), with continued recommendations that suicidality should be monitored when these medications are used (103). Common side effects include nausea, vomiting, diarrhea, headache, insomnia, and agitation (104). SSRI therapy has been reported to increase the risk of bleeding (platelet dysfunction related to the block of serotonin reuptake), most frequently during initiation of treatment, and particularly in the presence of concomitant aspirin or nonsteroidal anti-inflammatory agents (105). Therefore, clinicians should maintain awareness of the potential for unexplained bleeding, as well as the risk of prolonged QT interval (106).

## Conclusions

In light of the higher incidence and prevalence of mental health disorders in individuals with IBD, the adverse impact on disease course, the heightened healthcare utilization for those with IBD, and comorbid mental health concerns, there have been multiple calls for better integration of psychological and medical care in IBD clinics (80,107). Integrated care models, including specific examples such as the IBD medical home, are well established as the most effective approach. This approach not only provides a whole-person response to the management of IBD, which is valued by persons with IBD but also demonstrates a direct benefit to health systems through reduced IBD surgeries, hospital admissions and IBD comorbidities (107). The growing field of psychogastroenterology, which focuses on the brain-gut connection, the role of psychosocial factors, and the application of effective psychological approaches to gastrointestinal conditions, is well positioned to guide care models that optimize outcomes for individuals with IBD (80).

## Knowledge Gaps and Future Research Directions

1. Improving identification of those who are at risk for mental health concerns, especially during vulnerable periods around the IBD diagnosis and during transition from pediatric to adult care, will be important to ensure appropriate care is in place.
2. Little is known about the pathobiology of mental health disorders in IBD; however, there is increased attention to considerations of whether mood disorders are inflammatory diseases, and whether aspects such as fatigue in both IBD and depression potentially signal a cytokine imbalance as a common base. Understanding the pathobiology of mental health disorders in persons with IBD may lead to unique treatment approaches, including optimizing anti-inflammatory mechanisms of antidepressant medications.
3. Further research is needed into effective therapies for mental health concerns in those with IBD, examining impact on both mental health and IBD outcomes. IBD-tailored CBT and gut-directed medical hypnotherapy have growing evidence of positive effects for psychological outcomes for people with IBD, and potential benefits for IBD outcomes; this should be an area of focus for future studies.
4. Strengthening resilience to mitigate or even prevent stress-related disorders and improve outcomes for individuals with IBD is a promising line of inquiry, needing further longitudinal studies, ability to scale up interventions, and specific exploration for children and youth with IBD.
5. Virtual delivery of psychological therapies has been enabled by improved technological capabilities during the pandemic and has promise for broader accessibility to mental health care; these interventions need clinical assessment for efficacy and uptake among children and adults with IBD.
6. A better understanding of the potential mental health impact of caring for a person living with IBD of any age is needed to inform supportive resources for caregivers.

## Patient and Caregiver Partner Perspective

Patient partners expressed feelings of validation after reviewing this chapter, especially related to the identification of the bidirectional nature of mental health and disease course in IBD and the recognition that mental health concerns are common in persons living with IBD. Patient partners highly recommend that the gold standard in IBD management should include ongoing assessment and treatment for mental health concerns. By intervening early, health system costs related to hospitalizations, ED visits, and surgeries can be reduced. Psychological interventions (i.e., cognitive behavioural therapy, mindfulness, hypnotherapy) offer great promise to enhance the quality of life, mental wellness, and resilience of individuals living with IBD. However, it was noted that there were barriers to accessing mental health professionals and a lack of funding available to support access. Promising areas for future research noted by patient partners were on the topics of resilience, self-efficacy, and the links between gut and mental health.

## Policy Implications and Key Advocacy Outcomes

1. Considering the detrimental effects of IBD on the mental health of children and adolescents with IBD and their families, and the risk of long-term mental illness in these vulnerable individuals, a multidisciplinary team including mental health specialists should be available to all children and adolescents with IBD, optimally for prevention and early intervention.
2. Integrated care models for the adults with IBD, which incorporate mental health and medical services, are needed as the routine approach to care to benefit the individual and the health system.
3. Until integrated care models for IBD clinics are more readily available in jurisdictions across Canada, access to and funding for mental health care needs for children and adults with IBD should be prioritized.
4. Further research examining IBD outcomes for psychological and psychotropic medication therapies is needed to delineate the mechanisms and benefits of these mental health treatment approaches overall.
5. Enhanced physician education and resources to address mental health concerns are needed to facilitate more routine review and initiation of proper care pathways in the IBD clinic.
6. Enhancing mental health literacy for those with IBD, including children, adults, and their families, is important to facilitate identifying their care needs. Patient partners expressed that they knew how to describe their physical IBD symptoms, but sometimes lacked the language to raise or describe mental health concerns.

## Data Availability

No new data were generated or analyzed in support of this review.
